# Resistance to anti-PD-1/anti-PD-L1: galectin-3 inhibition with GB1211 reverses galectin-3-induced blockade of pembrolizumab and atezolizumab binding to PD-1/PD-L1

**DOI:** 10.3389/fimmu.2023.1250559

**Published:** 2023-08-28

**Authors:** Joseph Mabbitt, Ian D. Holyer, James A. Roper, Ulf J. Nilsson, Fredrik R. Zetterberg, Lynda Vuong, Alison C. Mackinnon, Anders Pedersen, Robert J. Slack

**Affiliations:** ^1^Stevenage Bioscience Catalyst, Galecto Biotech AB, Stevenage, United Kingdom; ^2^Nine Edinburgh BioQuarter, Galecto Biotech AB, Edinburgh, United Kingdom; ^3^Department of Chemistry, Lund University, Lund, Sweden; ^4^Sahlgrenska Science Park, Galecto Biotech AB, Gothenburg, Sweden; ^5^Department of Surgery, Urology Service, Memorial Sloane Kettering Cancer Centre, New York, NY, United States; ^6^Cobis Science Park, Galecto Biotech AB, Copenhagen, Denmark

**Keywords:** PD-1 - PDL-1 axis, galectin-3 (Gal3), atezolizumab, pembrolizumab, computational chemistry, immuno-oncology, binding

## Abstract

**Background:**

Galectin-3 (Gal-3) is a β-galactoside-binding lectin that is highly expressed within the tumor microenvironment of aggressive cancers and has been suggested to predict a poor response to immune checkpoint therapy with the anti-PD-1 monoclonal antibody pembrolizumab. We aimed to assess if the effect of Gal-3 was a result of direct interaction with the immune checkpoint receptor.

**Methods:**

The ability of Gal-3 to interact with the PD-1/PD-L1 complex in the absence and presence of blocking antibodies was assessed in *in vitro* biochemical and cellular assays as well as in an *in vivo* syngeneic mouse cancer model.

**Results:**

Gal-3 reduced the binding of the checkpoint inhibitors pembrolizumab (anti-PD-1) and atezolizumab (anti-PD-L1), by potentiating the interaction between the PD-1/PD-L1 complex. In the presence of a highly selective Gal-3 small molecule inhibitor (GB1211) the binding of the anti-PD-1/anti-PD-L1 therapeutics was restored to control levels. This was observed in both a surface plasmon resonance assay measuring protein-protein interactions and *via* flow cytometry. Combination therapy with GB1211 and an anti-PD-L1 blocking antibody reduced tumor growth in an *in vivo* syngeneic model and increased the percentage of tumor infiltrating T lymphocytes.

**Conclusion:**

Our study suggests that Gal-3 can potentiate the PD-1/PD-L1 immune axis and potentially contribute to the immunosuppressive signalling mechanisms within the tumor microenvironment. In addition, Gal-3 prevents atezolizumab and pembrolizumab target engagement with their respective immune checkpoint receptors. Reversal of this effect with the clinical candidate GB1211 offers a potential enhancing combination therapeutic with anti-PD-1 and -PD-L1 blocking antibodies.

## Background

Galectin-3 (Gal-3) is a β-galactoside-binding mammalian lectin. It is the only chimeric galectin with a C-terminal carbohydrate recognition domain (CRD) linked to a proline, glycine, and tyrosine rich additional N-terminal domain (1). Gal-3 binds several cell surface glycoproteins *via* its CRD domain and undergoes oligomerization, *via* binding at the N-terminal CRD domain, resulting in the formation of a Gal-3 lattice on the cell surface (2–4). The Gal-3 lattice has been regarded as being a crucial mechanism whereby extracellular Gal-3 modulates cellular signalling by prolonging retention time or retarding lateral movement of cell surface receptors in the plasma membrane. As such, Gal-3 can regulate various cellular functions such as diffusion, compartmentalisation and endocytosis of plasma membrane glycoproteins and glycolipids, and the functionality of membrane receptors (5).

Gal-3 has been shown to affect multiple processes in the tumor microenvironment (TME), such as promoting tumor invasion, migration, and angiogenesis (6), and has immunosuppressive effects due to promoting M2 macrophage activation and inhibiting T cell function (7–9). Gal-3 is highly expressed across many cancers (10) with high expression associated with poor response to pembrolizumab (11) and resistance to platinum-based chemotherapy in non-small cell lung cancer (NSCLC, 12). Gal-3 is a recognised ligand of the lymphocyte activation gene-3 (LAG-3), an immune checkpoint receptor expressed on the surface of effector T cells and regulatory T cells, and associated with worse prognosis in several cancers (13). In addition, it has also been shown in NSCLC models that inhibition of Gal-3 enhances the performance of immune checkpoint inhibitors (ICIs) targeting the programmed cell death 1/programmed cell death 1 ligand (PD-1/PD-L1) axis (14, 15). However, the mechanism by which Gal-3 negatively impacts the effect of PD-1/PD-L1 based checkpoint inhibitors is not fully understood.

In this study, we investigated the hypothesis that Gal-3 interacts directly with the PD-1/PD-L1 complex itself preventing the binding and activity of immune checkpoint inhibitors. This approach has not been investigated before and would potentiallysuggest a novel and unique mechanism of action (MOA) for Gal-3 inhibitors on enhancing antitumor immunity with checkpoint inhibitors.Further characterisation of this potential MOA was performed *in vitro* by investigating the effect of GB1211, a Gal-3 selective small molecule inhibitor currently in clinical development (16) as a combination therapy with atezolizumab in patients with advanced NSCLC (NCT05240131), on reversing Gal-3 binding to PD-1 and PD-L1 in the absence and presence of the ICIs. In addition, *in vivo* studies were also completed investigating GB1211 effects on promoting regression of tumor growth of subcutaneously transplanted Lewis lung carcinoma cells (LLC1) in C57Bl/6 mice, with or without combined treatment with an anti-PD-L1 blocking antibody.

## Methods

### Materials

PD-1 (10084-H08H) and PD-L1 (10377-H08H) human recombinant proteins were purchased from Sino Biological (China). Jurkat-Lucia™ TCR-hPD-1 and Raji-APC-hPD-L1 were purchased from InvivoGen (France). Human recombinant Gal-3 was generated as detailed previously (17). The human monoclonal antibodies atezolizumab and pembrolizumab were purchased from MedChemExpress (USA). GB1211 was synthesised by the Galecto Biotech AB Medicinal Chemistry Department (Sweden).

### Equipment and software

**Figure 1:**
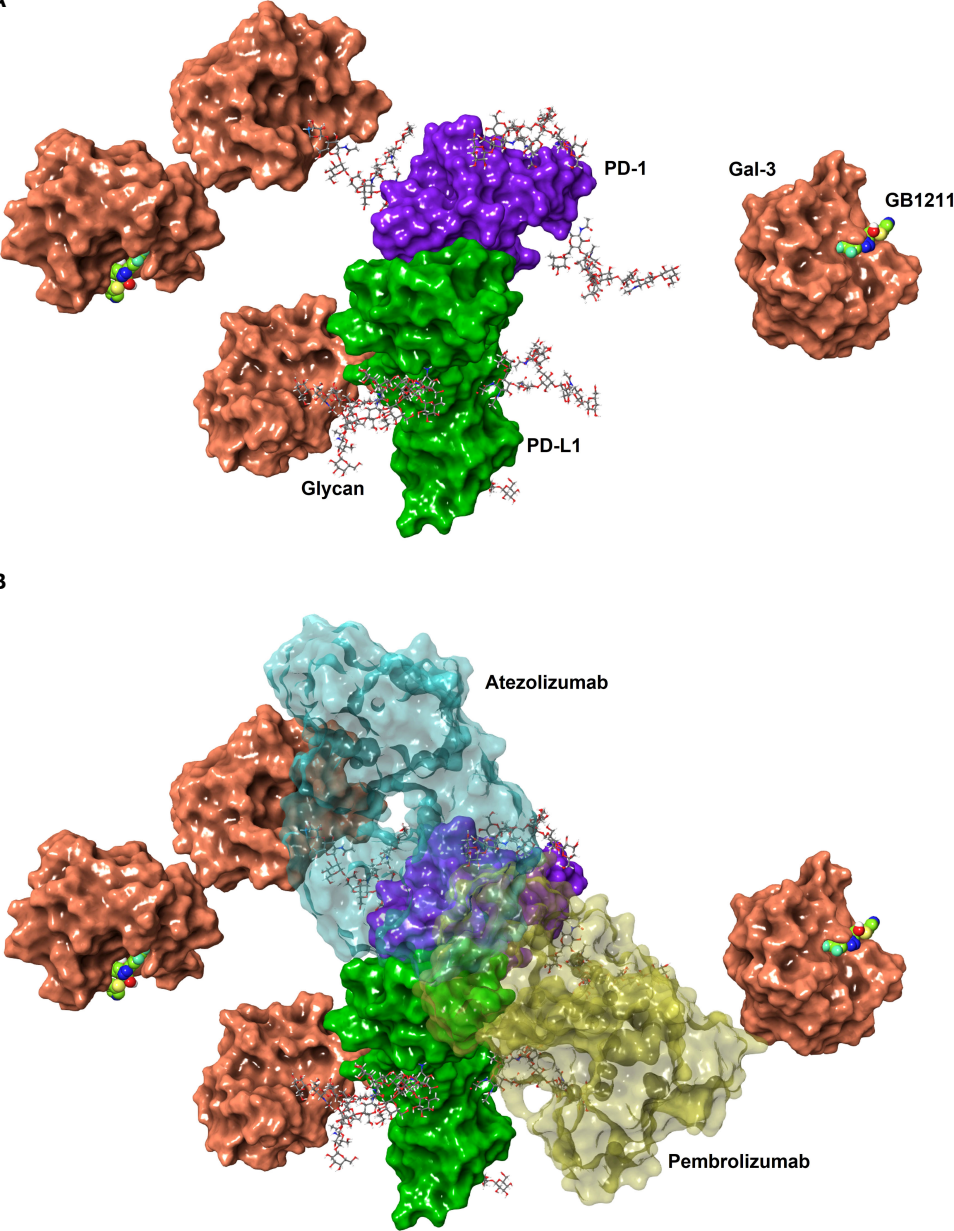
**(A)** Connolly surfaces of PD-1 (purple) binding PD-L1 (green) carrying four N-glycans (sticks) each at the known N-glycan sites for PD-1 (N49, N58, N74 and N116. (18) and PD-L1 (N35, N192, N200, and N219 (19). Gal-3 is shown as a Connolly surface (salmon) bound to a terminal D-galactoside residues of the PD-1 N58 and PD-L1 N192 glycans or to the inhibitor GB1211 (space-filling atoms with green carbons). The glycans were built manually onto the X-ray structures of full-length PD-L1 (4Z18) and on PD-1 in the PD-1/PD-L1 complex (4ZQK), whereafter the structures were superposed to show a possible binding of full-length PDL1 to PD1. The X-ray structure of Gal-3 in complex with GB1211 (7ZQX) was used to visualise inhibited Gal-3 and to manually superpose Gal-3 to the glycoprotein N-glycans. **(B)** The overlay of the binding interface between pembrolizumab (yellow) to PD-1 and atezolizumab (cyan) to PD-L1 and are shown as transparent surfaces for reference. The structures and interaction positions of pembrolizumab and atezolizumab were taken from their X-ray structures with PD-1 (5GGS) and PD-L1 (5XXY), respectively. Note, glycan structures are generic branched complex N-glycans and may not represent the most prevalent structures experimentally determined. The glycans were energy minimised to represent possible minimum conformations, but not necessarily the global minimum conformations.

Surface plasmon resonance (SPR) was completed using a Biacore™ T200 or Biacore™ 8K+ and data analysed using either the Biacore™ T200 Evaluation Software, Biacore™ T200 Control Software, Biacore™ 8K+ Control Software or Biacore™ 8K+ Insight Evaluation Software (all Cytiva, USA)). Flow cytometry was completed using a CytoFLEX and analysed on the CytExpert software (both Beckman Coulter Life Sciences, USA). Data visualisation and statistical analysis were completed using GraphPad Prism 9.3.3. The structures in **Figure 1** were built in Schrödinger Maestro (Release 2022-4: Maestro, Schrödinger, LLC, New York, NY, 2021).

### Cell culture

Jurkat-Lucia™ TCR-hPD-1cells and Raji-APC-hPD-L1 cells were cultured at 37°C (5% CO_2_) in Iscove’s Modified Dulbecco’s Medium (IMDM) containing 2mM L-glutamine, 25 mM 4-(2-hydroxyethyl)-1-piperazineethanesulfonic acid (HEPES), 10% heat inactivated fetal bovine serum, 100 U/mL penicillin, 100 μg/mL streptomycin and either 100 μg/mL Normocin™ or 10 μg/mL Blasticidin, respectively, then plated at 80,000 cells per well in a 96-well TC treated V-bottomed plate. LLC1 cells expressing Gaussia princeps luciferase were purchased and cultured as described (14).

### General SPR binding methods

Immobilisation pH scouting was performed prior to immobilisation of proteins to determine the optimised pH for capture of a ligand on a carbomethyl dextran CM5 sensor chip. Ligand was diluted in acetate buffers of pH 4, 4.5, 5, 5.5 before being associated over a fresh CM5 sensor chip to determine the slope and stability of capture. The surface was then regenerated using a 30 s 5 μL/mL injection of 50 mM NaOH. For analysis, either a 1:1 fitting was used with appropriate reference subtraction, or the Rmax was used. For the generation of the % binding over time, the export of curve timepoints was used and referenced to the theoretical Rmax based on displaced binding. Full details of SPR methods covering GB1211 binding to Gal-3 and Gal-3 binding to PD-1 and PD-L1 (alone and in combination) in the absence and presence of inhibitors can be found in the Supplementary section.

### Cell surface antibody binding *via* flow cytometry

Plated Raji-APC-hPD-L1 cells or Jurkat-Lucia™ TCR-hPD-1 cells were blocked with IMDM containing 2% bovine serum albumin (blocking buffer) for 20 min then media replaced with 100 μL IMDM containing titrated atezolizumab (Raji-APC-hPD-L1 cells) or pembrolizumab (Jurkat-Lucia™ TCR-hPD-1 cells) in the presence or absence of either 2 μM Gal-3, 500 nM PD-1 (Raji-APC-hPD-L1 cells) or PD-L1 (Jurkat-Lucia™ TCR-hPD-1 cell), 2μM GB1211 or combinations thereof. Cells were incubated for 1 h then washed with blocking buffer and stained with annexin-V-APC and an anti-IgG1 (Raji-APC-hPD-L1 cells) or anti-IgG4 (Jurkat-Lucia™ TCR-hPD-1 cells) FITC antibody diluted 1:600 for 30 min. All wells were then washed and fixed using 4% paraformaldehyde. Plates were then analysed on the CytoFLEX and single cells gated using a polygonal gate to determine the cell population using FSC-A and FSC-H. Single live cells were then identified as annexin-V negative. FITC was used to determine binding of atezolizumab or pembrolizumab through the conjugated anti-IgG1 or anti-IgG4, respectively. % of live single cells positive for FITC were then gated based on the minimum and maximum atezolizumab or pembrolizumab binding.

### *In vivo* experimental protocol

All animal experimental work was carried out under a project license approved by the local Animal Welfare and Ethical Review body (AWERB) and issued in accordance with the Animals (Scientific Procedures) Act 1986. C57Bl/6 mice were purchased from Harlan Laboratories. LLC1 cells were injected subcutaneously into the flanks of male C57Bl/6 mice. Each animal received an injection of 2.5 x 10^5^ cells suspended in 100 μL phosphate buffered saline (PBS) in both flanks. Tumor volumes were measured with digital callipers every 1-3 days. Tumor volume was calculated according to the formula:


$$\text{Tumor}\;\text{volume}\;\left(\text{m}{\text{m}}^{3}\right)\;=\;\text{π}/6\;\times \;{\left(\text{length}\;\times \;\text{width}\right)}^{3/2}$$

### Drug administration

GB1211 for oral administration was prepared in poly ethylene (PEG) 300/Solutol HS 15 (90/10% v/v) at a concentration of 1 mg/mL and mice received 10 mL/kg dose volume equivalent to a 10 mg/kg dose twice daily by oral gavage. The anti-PD-L1 monoclonal antibody (clone 10F.9G2) was purchased from BioXCell and 200 μg in PBS was administered twice weekly by i.p. injection commencing on day 6 for a total of 4 injections.

### Tumor digests and flow cytometry

Tumors were minced in serum-free Dulbecco’s Modified Eagle Medium and digested with Liberase (2 mg/ml; Sigma-Aldrich, USA) and DNase I (Sigma-Aldrich, USA) at 37°C for 30 mins. Disaggregated tissue was filtered through a 35 μm nylon mesh, washed and resuspended in fluorescence-activated cell sorting (FACS) buffer (PBS with 0.1% bovine serum albumin). Fc receptors were blocked with anti-mouse CD16/32 (Biolegend, USA). Antibody cocktails (anti-mouse CD45-PerCP, CD3-PerCPcy5.5, CD8-AF700, all from Biolegend, USA) were added to cells (1 μL per test) and incubated for 20 mins at room temperature. Samples were washed x 2 in FACS buffer, fixed and red blood cells (RBCs) were simultaneously lysed in RBC Lysis/Fixation solution (Biolegend, USA). For intracellular staining, cells were permeabilized with intracellular staining permeabilization wash buffer (Biolegend, USA) and incubated with anti-Ki67-PE (Biolegend, USA). Cells were collected using an LSR-Fortessa cell analyser (Beckton Dickenson, USA) and analysed using FlowJo software, version 10 (Tree Star Inc., USA).

### Statistical analysis

Statistical analyses were performed using GraphPad Prism 9.0 software. Results are represented as mean ± SEM and statistical tests are described in the figure legends, where applicable.

## Results

### Gal-3 potentiates PD-1/PD-L1 binding and is reversed by GB1211

**Figure 2:**
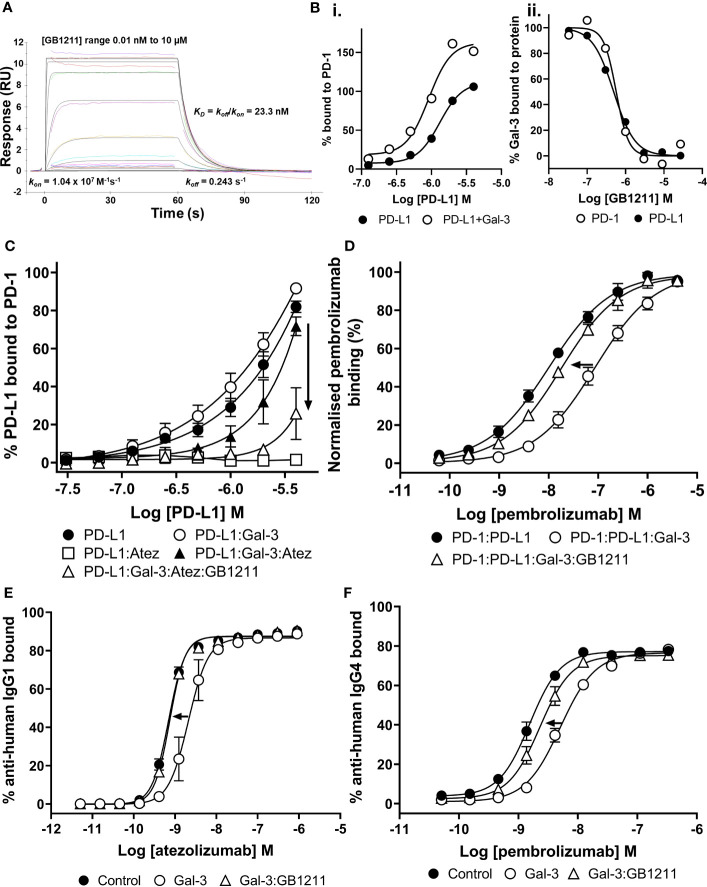
**(A)** Profile of GB1211 binding to Gal-3 as measured by SPR. **(B)** Gal-3 potentiates the binding of PD-L1 to PD-1 with a 3-fold increase in affinity. (Bii) GB1211 blocks the binding of Gal-3 (1 µM) to immobilised PD-1 and PD-L1 with an IC_50_ of 0.57 µM and 0.50 µM, respectively. **(C)** Gal-3 potentiates the binding of PD-L1 to immobilised PD-1 that reduces the ability of atezolizumab to bind the PD-1/PD-L1 complex. GB1211 reverses the Gal-3 blockade restoring the binding of atezolizumab to PD-L1. **(D)** Gal-3 reduces the binding of pembrolizumab to immobilised PD-1 that is reversed by GB1211. **(E)** Atezolizumab binding to PD-L1 on the surface of Raji-hPD-L1 cells measured by flow cytometry. Gal-3 reduces the ability of atezolizumab to bind to the Raji-hPD-L1 cells, with the Gal-3 inhibitor GB1211 able to reverse this blockade. **(F)** Pembrolizumab binding to PD-1 on the surface of Jurkat-hPD-1 cells measured by flow cytometry. Gal-3 reduces the ability of pembrolizumab to bind to the Jurkat-hPD-1 cells, with the Gal-3 inhibitor GB1211 able to reverse this blockade. Data shown is mean ± SEM (n=3) with arrows showing the restorative effect of GB1211 on ICI. Atez, atezolizumab.

GB1211 bound to Gal-3 with high affinity (kinetic K_D_ = 23.3 nM (*k_on_*/*k_off_*)) with a fast association (*k_on_*) and fast dissociation rate (*k_off_*) (**Figure 2A**). From SPR kinetic analysis of PD-L1 binding to PD-1 in the absence and presence of Gal-3 (**Figure 2B**i), a 3-fold increase in affinity was observed for PD-L1 in the presence of Gal-3 (kinetic K_D_ of 1.1 μM in presence of Gal-3 *vs.* 3.3 μM in the absence of Gal-3). GB1211 inhibited the Gal-3 binding to both PD-1 and PD-L1 with an IC_50_ of 0.57 μM and 0.49 μM, respectively (**Figure 2B**ii). This data shows that Gal-3 promotes the PD-1/PD-L1 interaction and this is inhibited with GB1211.

### Gal-3 reduces atezolizumab and pembrolizumab affinity for their checkpoint receptors that is reversible with GB1211

We next evaluated atezolizumab’s interaction with the PD-1/PD-L1 complex. As expected atezolizumab completely inhibited the binding of PD-1 with PD-L1. This interaction was inhibited in the presence of Gal-3 (**Figure 2C**). GB1211 was shown to reverse the Gal-3 effect by restoring the binding of atezolizumab to PD-L1. In experiments measuring the impact of Gal-3 on pembrolizumab’s interaction with the PD-1/PD-L1 complex, Gal-3 reduced the ability of pembrolizumab to bind to the PD-1/PD-L1 complex (5.8-fold shift). GB1211 was again shown to reverse the Gal-3 effect by restoring the binding of pembrolizumab to PD-1 (**Figure 2D**). These data suggest that Gal-3 can inhibit the interaction of ICIs with their immune checkpoints and that GB1211 can restore effective ICI binding. Using Raji-APC-hPD-L1 cells, it was again evident that Gal-3 can also reduce the ability of atezolizumab to bind to PD-L1 on the cell surface (3.7-fold shift in EC_50_) (**Supplementary Table 1**). GB1211 was shown to reverse the Gal-3 effect by restoring the binding of atezolizumab (**Figure 2E**). The impact of Gal-3 on pembrolizumab’s interaction with the PD-1/PD-L1 was examined on Jurkat™-Lucia TCR-hPD-1 cells, it was again evident that Gal-3 reduced the ability of pembrolizumab to bind to PD-1 on the cell surface (3.2-fold shift in EC_50_) (**Supplementary Table 1**). GB1211 reversed the Gal-3 effect by restoring the binding of pembrolizumab to Jurkat™-Lucia TCR-hPD-1 cells (**Figure 2F**). The hypothesized interactions, derived from X-ray structures, between Gal-3 and the key glycans in proximity to the binding sites between PD-1/PD-L1, pembrolizumab/PD-1 and atezolizumab/PD-L1 are summarised in **Figure 1**.

### GB1211 facilitates anti-PD-L1 inhibition of tumor growth *in vivo*

**Figure 3:**
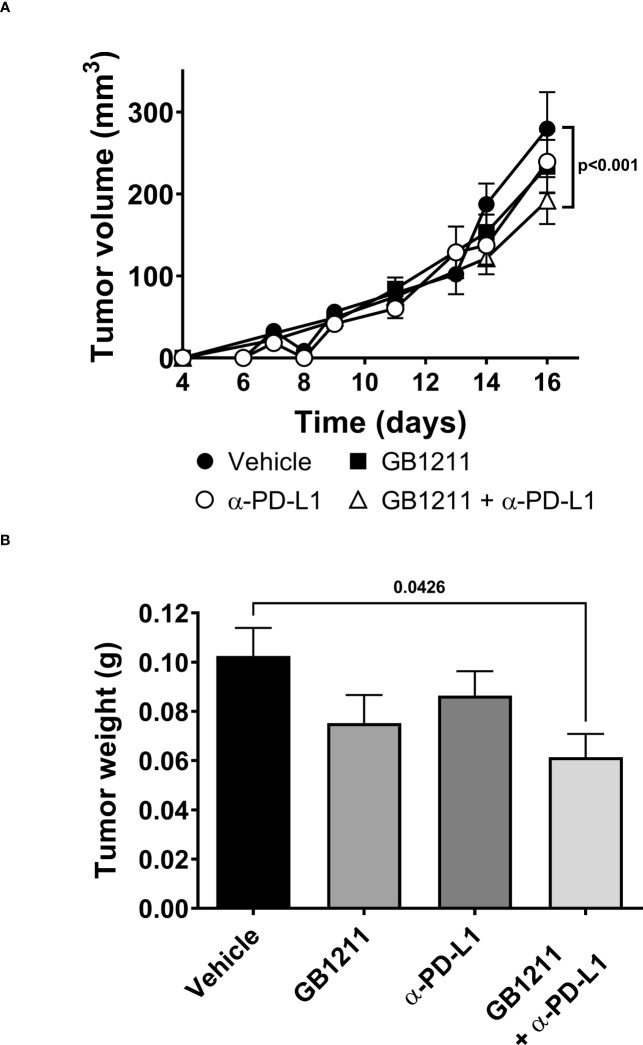
LLC1 subcutaneous tumor **(A)** volumes and **(B)** weights. Results represent the mean ± SEM (vehicle n=28 tumors from n=16 mice, GB1211 (10 mg/kg) n=12 tumors from n=8 mice, anti-PD-L1 (200 µg) n=28 tumors from n=16 mice, GB1211 (10 mg/kg) + anti-PD-L1 (200 µg) n=14 tumors from n=8 mice). Two-way ANOVA with Tukey’s *post-hoc* test was used to test for differences in tumor volume with only significant changes shown. One-way ANOVA and Dunnett’s post-test were used to compare tumor weight with only significant changes shown.

**Figure 4:**
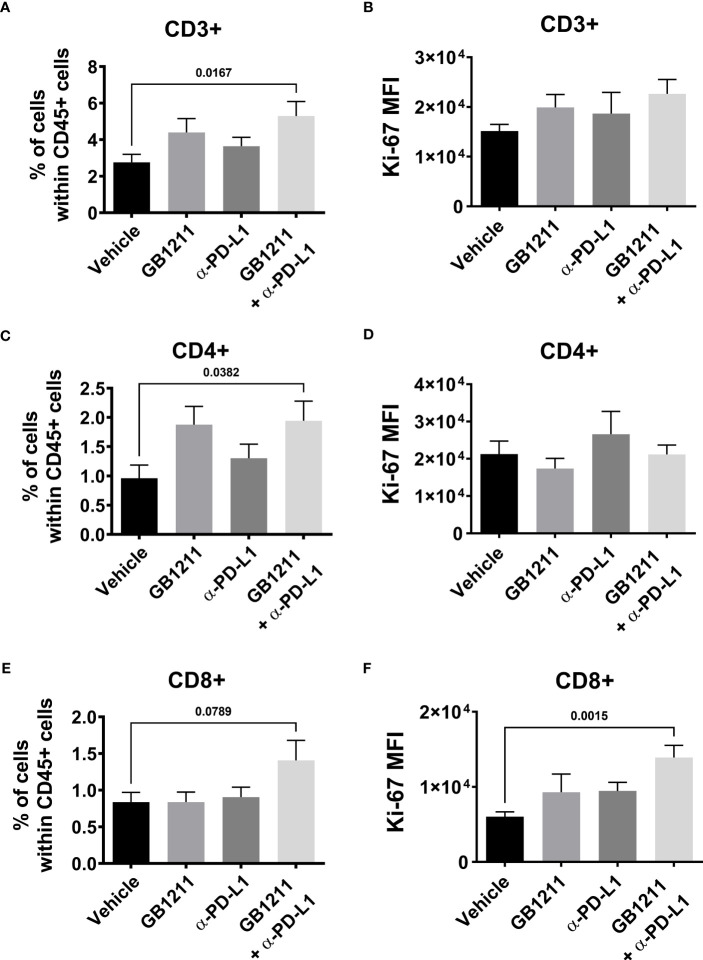
*post-hoc*LLC1 subcutaneous tumor digests were analysed for tumor infiltrating CD8+ T cell populations by flow cytometry. Frequency of **(A)** CD3+, **(C)** CD4+ and **(E)** CD8+ cells expressed as a % of the total immune (CD45+) population and Ki-67 expression as mean fluorescence intensity (MFI) in **(B)** CD3+, **(D)** CD4+ and **(F)** CD8+ T cells. Results are expressed as mean ± SEM (vehicle n=12, GB1211 n=10, anti-PD-L1 n=10, GB1211+anti-PD-L1 n=12). Analysed *via* one-way ANOVA with Dunnett’s post-test with only significant changes shown except for drug combination p-value shown in panel 4 **(E)**.

We examined the impact of single and combination therapy with GB1211 (10 mg/kg, twice daily orally) and anti-PD-L1 treatment (200 mg *i.p.* twice weekly for 4 administration) in a syngeneic mouse LLC1 lung adenocarcinoma model that has been shown to be resistant to anti-PD-L1 therapeutic antibodies (20–22). Treatment commenced on day 6 post-tumor cell inoculation when tumors were just palpable in the vehicle treatment group. Anti-PD-L1 or GB1211 administration as monotherapies resulted in a non-significant reduction in tumor growth.Following combination treatment ofGB1211 and anti-PD-L1 antibody final tumor volume and weight (31.3% and 40.1% reduction, respectively) were significantly reduced compared with vehicle control (**Figures 3A, B**, respectively). The reduced tumor growth in the combination group was also associated with a trend or significant increase in the % of tumor infiltrating CD3+, CD4+ and CD8+ T cells (**Figures 4A, C, E**, respectively) and an increase in the proliferation index (Ki-67 expression) of CD3+ and CD8+ T cell subsets (**Figures 4B, F**, respectively). There were no effects on the proliferation index of CD4+ T-cells (**Figure 4D**) or any other key immune cell populations measured (**Supplementary Figure 1**).

## Discussion

**Figure 5:**
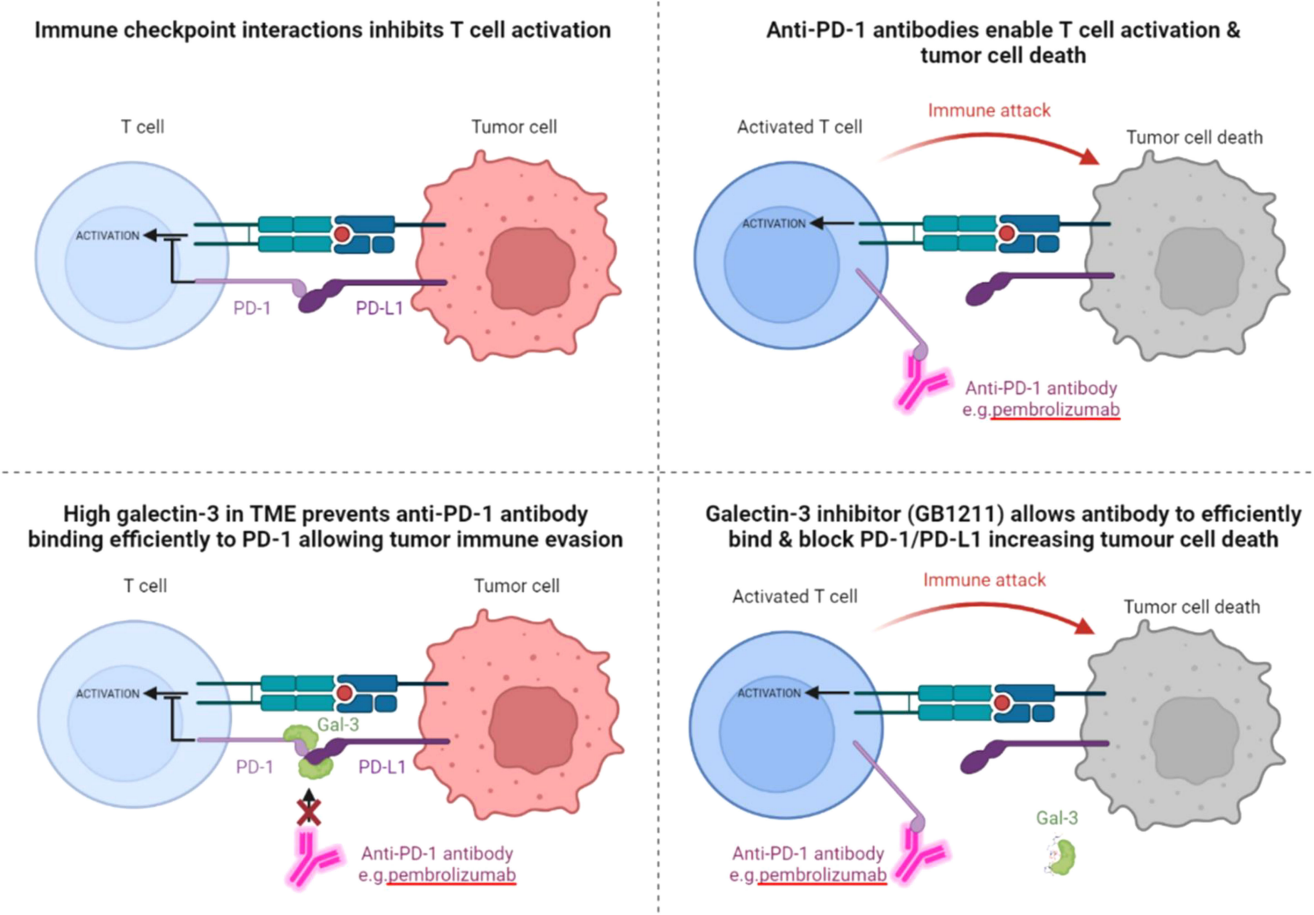
Schematic depicting the relationship between Gal-3 and the checkpoint receptor PD-1, with the proposed negative effect of high Gal-3 on pembrolizumab binding leading to blockade of the ICI’s T cell activation and tumor suppression effects. The proposed reversal of Gal-3’s inhibitory effect on pembrolizumab’s target engagement is also shown with the Gal-3 inhibitor GB1211. The same effect on atezolizumab and PD-L1 is also proposed from the data in this study.

High Gal-3 expression in several cancers has been suggested to predict a poor response to immune checkpoint therapy with the anti-PD-1 monoclonal antibody pembrolizumab, including human lung adenocarcinoma and melanoma patients (11, 23). We sought to investigate the mechanism whereby Gal-3 confers resistance to ICI therapy. Our earlier work has shown that genetic deletion of Gal-3 in the host reduces lung cancer growth in the mouse syngeneic LLC1 lung adenocarcinoma model (14). Treatment with the Gal-3 inhibitor GB1107 – a GB1211 analogue - potentiated the response to anti-PD-L1 and increased infiltration and proliferation of CD8^+^ T cells and T cell derived cytotoxic mediators (IFNγ, perforin-1, granzyme B, and fas ligand (14)). However, the mechanism of action of GB1107 to potentiate ICI responses was not completely understood. In another study GB1107 was shown to increase the response to anti-PD-L1 therapy in a human xenograft model of NSCLC following transplant with human peripheral blood monocytic cells (PBMCs) (15). In the current study, Gal-3 was shown to vastly reduce the binding of the checkpoint inhibitors pembrolizumab and atezolizumab, in part by potentiating the interaction between PD-1 and PD-L1, an effect that was reversed by GB1211. This strengthens the scientific rationale that Gal-3 confers resistance to ICI and blocking Gal-3 with GB1211 restores response to ICI therapy. A proposed summary of this interaction is depicted in **Figure 5**, with pembrolizumab’s interaction with PD-1 in the absence and presence of Gal-3, GB1211 or combinations thereof, used as the example ICI/checkpoint receptor pairing. GB1211 has demonstrated high human Gal-3 affinity and selectivity versus other members of the galectin family investigated (24). GB1211 is also orally active and so has advantages over other putative Gal-3 inhibitors such as belapectin and GCS-100 (25) which are large complex carbohydrates with poor bioavailability and selectivity (26, 27). In this study, GB1211 restored the binding of the anti-PD1/anti-PDL1 therapeutics and may thus reduce tumor resistance to these agents, especially in cancers expressing high levels of Gal-3. Interestingly, it has been shown that atezolizumab’s affinity is negatively impacted when glycans are removed from PD-L1 (Benicky et al, 2021). This supports the observations here whereby Gal-3 binding to glycans on PD-L1 would result in a decrease in the affinity of atezolizumab for its receptor. This has also been demonstrated for the PD-1 antibody camrelizumab (28), where the affinity of the ICI is substantially reduced for glycosylation-deficient PD-1. There is also increasing literature demonstrating the key role of N-linked glycosylation states of the PD-1 and PD-L1 in cancer and the potential response to therapies (29–31).

It is worth highlighting that the PD-1 and PD-L1 proteins used in this study are the native, recombinantly produced, non-disease forms. As the field of understanding has built in respect to glycan profiles and their influence in the TME, it is now well established that shifts in cellular glycosylation patterns are a hallmark of cancer (32). Therefore, it could also be hypothesised that the effect of Gal-3 on checkpoint inhibitors, as well as other mechanisms, would be greater in a glycan rich TME compared to the *in vitro* setting investigated in this study. This would suggest that the effect of GB1211 in an *in vivo* TME setting could be much more impactful. Although not investigated in this study, it could also be hypothesised that with this seismic shift in the glycan profiles in cancer, as well as other disease settings, that Gal-3 and other galectins could be responsible for the resistance of other therapies *via* enhanced interactions with a range of pro-cancer receptors.

Combination therapy with GB1211 and an anti-PD-L1 blocking antibody reduced *in vivo* tumor growth in the LLC1 syngeneic model of lung adenocarcinoma. An increased percentage and proliferation of tumor infiltrating T lymphocytes, particularly CD8 + T cells, was also observed using this combination approach, effects which were in concordance with previous studies with other Gal-3 inhibitors (14). This is a key characteristic of ICIs, as single therapies, when tumor killing efficacy is observed in the clinic. These findings compliment the additional *in vivo* tumor model investigations and clinical data reports showing an association between Gal-3 expression and lack of efficacy of PD-1/PD-L1 targeted checkpoint inhibitors (11, 14). GB1211 is currently in a phase II trial to evaluate the safety and efficacy in combination with atezolizumab in NSCLC (NCT05240131), with additional trials planned to start in 2023 in combination with pembrolizumab in HNSCC and melanoma (33). Although the main mechanism of action investigated here focuses on Gal-3 as a scaffold for PD1/PDL1 interactions which prohibits ICIs to bind and cause T-cell activation, Gal-3 may also contribute to additional TME immune suppressive mechanisms that minimise the current efficacy observed with ICIs in the clinic. An example of this is the suggestion that Gal-3 can trap IFNγ in the tumor stroma thus preventing the recruitment and activation of cytotoxic T cells (CTL) while blockade of Gal-3 causes an increase in cytokine activity which increased the recruitment of CTLs into the tumor (34). In theory, ICIs would then be able to activate these additional entities at the site of action which may increase tumor killing. GB1107 was shown to increase IFNγ expression in LLC1 tumors alone and potentiate expression in combination with an anti-PD-L1 (14). Gal-3 may also engage additional checkpoints and suppress CTL function by binding to LAG-3 (35) and promote the activation of immunosuppressive tumor associated macrophages (TAMs) (7, 14). TAMs promote many important features of tumor progression including angiogenesis, tumor cell invasion and metastasis and also suppress T cell responses *via* PD-L1 (36). Gal-3 expressing TAMs develop an M2 phenotype and downregulate of CD8+ CTL function whereas inhibition of Gal-3 reprograms the TME to favour proinflammatory M1-like macrophages and enhanced CTL function (14). Gal-3 has also been shown to effect other immunosuppressive mechanisms within tumors. For example, Gal-3 inhibition plus anti-OX40 therapy reduces monocytic myeloid suppressor cell (M-MDSC)-meditated immune suppression thereby increasing CD8+ T cell recruitment leading to increased tumor regression (37).

Collectively, our study suggests that Gal-3 can potentiate the PD-1/PD-L1 immune axis and contribute to the immunosuppressive signalling mechanisms within the TME. In addition, Gal-3 prevents atezolizumab and pembrolizumab target engagement with their respective immune checkpoint receptors, that can be reversed with the clinical Gal-3 inhibitor GB1211, offering a potential enhancing combination therapeutic with anti-PD-1 and -PD-L1 blocking antibodies.

## Data availability statement

The raw data supporting the conclusions of this article will be made available by the authors, without undue reservation.

## Ethics statement

Ethical approval was not required for the studies on humans in accordance with the local legislation and institutional requirements because only commercially available established cell lines were used. The animal study was approved by All animal experimental work was carried out under a project license approved by the local Animal Welfare and Ethical Review body (AWERB) and issued in accordance with the Animals (Scientific Procedures) Act 1986. The study was conducted in accordance with the local legislation and institutional requirements.

## Author contributions

FZ, UN - design and synthesis of selective Galectin-3 inhibitor (GB1211) UN, AP, FZ - structure-based computational binding analysis JM, JR - *in vitro* methodology development JM, RS, JR - *in vitro* data acquisition LV, AM, IH - *in vivo* data acquisition (design, implementation and post analysis) JM, IH, LV, AM, RS - analysis and interpretation of data (e.g., statistical analysis, biostatistics) IH - writing of manuscript IH, RS, FZ,UN, AM, AP - review and/or revision of the manuscript. All authors contributed to the article and approved the submitted version.

## Supplementary material

Supplementary Figure 1 LLC1 subcutaneous tumor digests were analysed for **(A)** total macrophage **(B)** CD206+ macrophage, **(C)** CD206- macrophage and **(D)** neutrophil populations by flow cytometry. Frequency of cells expressed as a % of the total immune (CD45+) population. Results are expressed as mean ± SEM (vehicle n=12, GB1211 n=10, anti-PD-L1 n=10, GB1211+anti-PD-L1 n=12). Analysed *via* one-way ANOVA with Dunnett’s post-test with only significant changes highlighted.
