# Supplementary material for: Resistance to anti-PD-1/anti-PD-L1: galectin-3 inhibition with GB1211 reverses galectin-3-induced blockade of pembrolizumab and atezolizumab binding to PD-1/PD-L1
Source: Front Immunol. 2023 Aug 28;14:1250559. doi: 10.3389/fimmu.2023.1250559 (PMC10493609; doi:10.3389/fimmu.2023.1250559)
Supplement: Supplementary Figure 1 — LLC1 subcutaneous tumor digests were analysed for (A) total macrophage (B) CD206+ macrophage, (C) CD206- macrophage and (D) neutrophil populations by flow cytometry. Frequency of cells expressed as a % of the total immune (CD45+) population. Results are expressed as mean ± SEM (vehicle n=12, GB1211 n=10, anti-PD-L1 n=10, GB1211+anti-PD-L1 n=12). Analysed via one-way ANOVA with Dunnett’s post-test with only significant changes highlighted. [file DataSheet_1.pdf]

**SUPPLEMENTARY**

**SUPPLEMENTARY METHODS**

*Surface plasmon resonance (SPR) methods*

For measurement of GB1211 binding to Gal-3, a CM5 sensorchip was used to amine couple and immobilise Gal-3. EDC and NHS were mixed at a 50:50 ratio and associated over the flow cells for 240 s at a flow rate of 10 µL/min to activate the surface. Ligand was then flown over the chip at 10 µg/mL using the 'aim for' function to capture 650 RU of ligand. Flow cells were then deactivated with a 240 s association with 1M ethanolamine-HCL. Flow cell 1 was used as a reference with no ligand captured, the chip was then left for 12 h to equilibrate. To determine the kinetics of GB1211 binding the immobilised Gal-3, a titration of GB1211 added in running buffer (PBS P+, 0.2 M phosphate, 27 mM KCl, 1.5 M NaCl, 0.5% Tween20, 5% DMSO, 1 mM DTT at a pH 7). A multi-cycle approach was used, where after a baseline, compound was associated for 60 s at 30 µL/min followed by a 60 s dissociation at 30 µL/min. A regeneration was performed between each cycle involving a 200 s wash with running buffer to allow the analyte to fully dissociate.

For the measurement of Gal-3 potentiation of binding between PD-1 and PD-L1, a CM5 sensorchip was used to amine couple and immobilise PD-1. EDC and NHS were mixed at a 50:50 ratio and associated over the flow cells for 240 s at a flow rate of 10 µL/min to activate the surface. Ligand was then flown over the chip at 10 µg/mL using a 120 s association to capture approximately 70 RU of PD-1 on flow cells 1. Flow cells were then deactivated with a 240 s association with 1 M ethanolamine-HCL. A fixed concentration of 4 µM Gal-3 was used for all conditions with a titration of PD-L1 added. A baseline of 40 s was used with running buffer prior to a 120 s association with PD-L1 or PD-L1 + Gal-3 at 30 µL/mL. This was followed by a 60 s dissociation with running buffer. The response was plotted where full binding represented Gal-3 binding to immobilised ligand.

For measurement of Gal-3 binding to PD-1, PD-L1 and the PD-1/PD-L1 complex, a CM5 sensorchip was used to amine couple and immobilise PD-1 or PD-L1. EDC and NHS were mixed at a 50:50 ratio and associated over the flow cells for 240 s at a flow rate of 10 µL/min to activate the surface. Ligand was then flown over the chip at 10 µg/mL using a 120 s association to capture approximately 1000 RU of PD-1 or PD-L1 on flow cells 1 and 2, respectively. Flow cells were then deactivated with a 240 s

association with 1 M ethanolamine-HCL. A fixed concentration of 1 μM Gal-3 was used for all conditions with a titration of GB1211 tested in running buffer. A multi-cycle approach was used where each cycle was followed by a regeneration in running buffer. After a 40 s baseline in running buffer, the mixture of Gal-3 and titrated compound was associated for 20 s, followed by a 30 s dissociation. The response was plotted where full binding represented Gal-3 binding immobilised ligand. The data was normalised using the Biacore™ Insight Evaluation Software's EC50 function.

For measurement of the interaction between Gal-3, PD-1, PD-L1 and atezolizumab, a sensorchip was used to amine couple and immobilise PD-1. EDC and NHS were mixed at a 50:50 ratio and associated over the flow cells for 240 s at a flow rate of 10 μL/mL to activate the surface. Ligand was then flown over the chip at 10 μg/mL using a 120 s association to capture approximately 70 RU of PD-1 on flow cells 1 to 8 respectively. Flow cells were then deactivated with a 240 s association with 1 M ethanolamine-HCL. A multi-cycle approach using the A-B-A inject function was adopted to determine the effect of atezolizumab on preventing PD-L1 from binding the immobilised PD-1. The 'A' inject comprised of all components in the complex minus the addition of PD-L1. Gal-3 and GB1211 were added at a constant 4 μM. Inject 'B' comprised of the same analytes used in injection 'A' but with the addition of PD-L1 at 2 μM. Injection 'A' was performed for 30 s at 30 μL/mL followed by injection 'B' for 50 s at 30 μL/min. This was then followed by a further 'A' Inject for 50 seconds at 30 μL/min. Double reference subtraction was used against a blank for injection 'A' only. A regeneration step was completed after each cycle with addition of glycine pH 2.5 for 30 s. Report points from the sensorgram's saturated binding was used to determine a minimum and maximum pembrolizumab binding for normalisation of binding.

For measurement of the interaction between Gal-3, PD-1, PD-L1 and pembrolizumab, a CM5 sensorchip was used to amine couple and immobilise PD-1. EDC and NHS were mixed at a 50:50 ratio and associated over the flow cells for 240 s at a flow rate of 10 μL/min to activate the surface. Ligand was then flown over the chip at 10 μg/mL using a 120 s association to capture approximately 70 RU of PD-1 on flow cells 1 to 8 respectively. Flow cells were then deactivated with a 240 s association with 1 M ethanolamine-HCL. A multicycle A-B-A inject approach was used with injection 'A' comprised of either running buffer alone, PD-L1, Gal-3, PD-L1 and Gal-3, PD-L1, Gal-3 and GB1211 or Gal-3

and GB1211. Gal-3 and GB1211 added at a constant 4 μM and PD-L1 added at a constant 1 μM. Injection 'B' contained the same analyte as injection 'A' but with a titration of pembrolizumab added. A regeneration step was completed after each cycle with addition of glycine pH 2.5 for 30 s.

**SUPPLEMENTARY RESULTS**

**Table 1.** The reversal of Gal-3 blockade of atezolizumub binding to Raji-APC-hPD-L1 cells and pembrolizumab binding to Jurkat Lucia$^{TM}$ TCR-hPD-1 cells by GB1211.

| **Condition** | **Atezolizumab EC50 (nM)** | **Fold shift *vs.* control** |
|---|---|---|
| Atezolizumab (Control) | 2.7 ± 0.2 | - |
| Atezolizumab + Gal-3 | 10.1 ± 3.5 | 3.7 |
| Atezolizumab + Ga-l3 + GB1211 | 3.3 ± 3.1 | 1.2 |
| | | |
| **Condition** | **Pembrolizumab EC50 (nM)** | **Fold shift *vs.* control** |
| Pembrolizumab (Control) | 1.6 ± 0.4 | - |
| Pembrolizumab + Gal-3 | 5.1 ± 0.6 | 3.2 |
| Pembrolizumab + Gal-3 + GB1211 | 2.4 ± 0.4 | 1.5 |

Data shown is mean ± SEM from 3 experimental replicates.

**Supplementary Figure 1.**

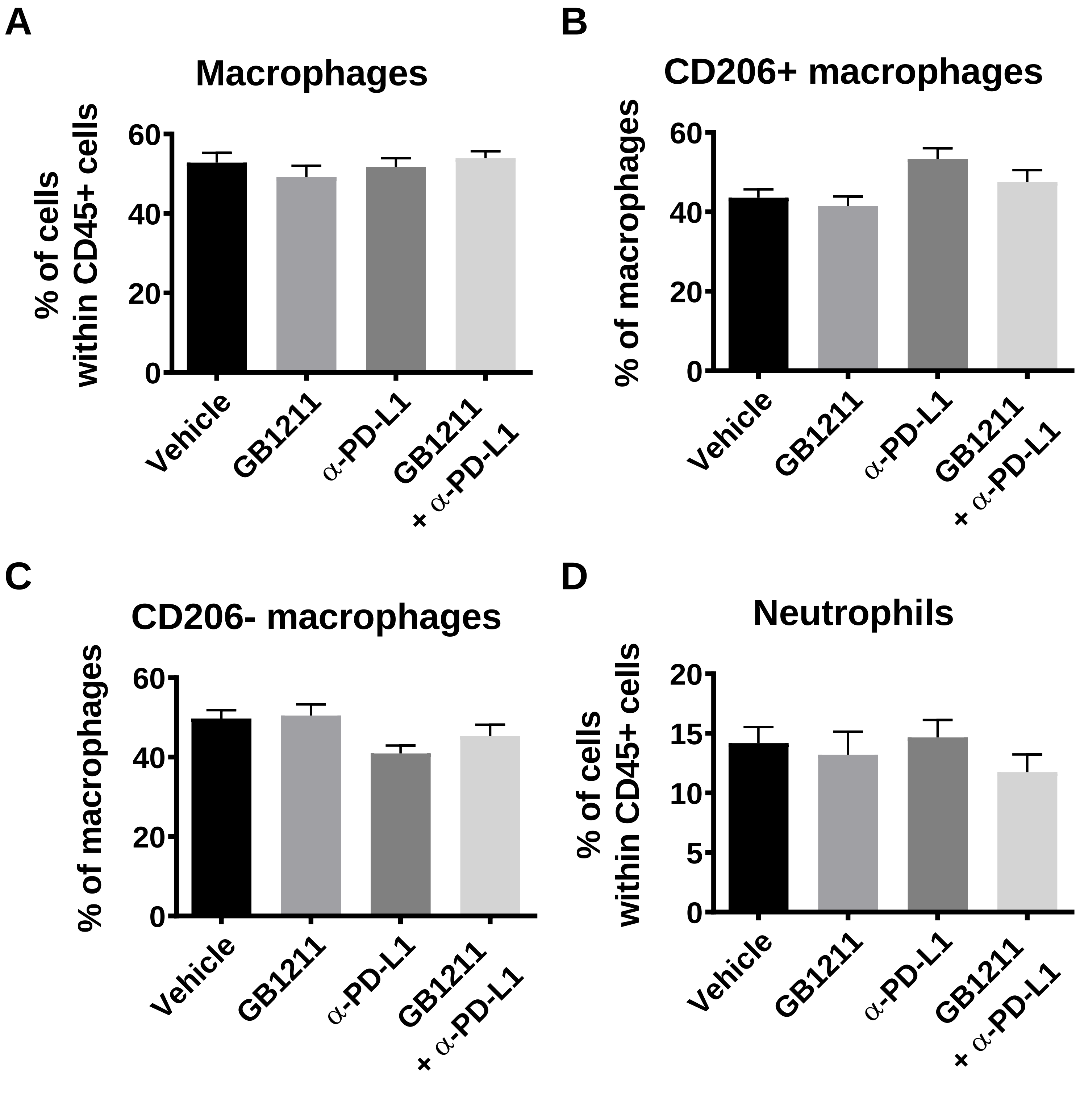

**Supplementary Figure 1.** LLC1 subcutaneous tumor digests were analysed for (A) total macrophage (B) CD206+ macrophage, (C) CD206- macrophage and (D) neutrophil populations by flow cytometry. Frequency of cells expressed as a % of the total immune (CD45+) population. Results are expressed as mean ± SEM (vehicle n=12, GB1211 n=10, anti-PD-L1 n=10, GB1211+anti-PD-L1 n=12). Analysed via one-way ANOVA with Dunnett's post-test with only significant changes highlighted.
